# Orthodontic appliances and their diagnostic impact to brain MRI

**DOI:** 10.1007/s00784-025-06275-8

**Published:** 2025-03-22

**Authors:** Lisa Latzko, Anna Schmit, Bernhard Glodny, Astrid E. Grams, Christoph Birkl, Adriano G. Crismani

**Affiliations:** 1 Department of Dental and Oral Medicine and Cranio-Maxillofacial and Oral Surgery, Medical University of Innsbruck, Innsbruck, Austria; 2 Department of Radiology, Medical University of Innsbruck, Anichstraße 35, 6020 Innsbruck, Austria; 3 Neuroimaging Core Facility, Medical University of Innsbruck, Innsbruck, Austria

**Keywords:** Orthodontic Appliances, Magnetic Resonance Imaging, Brain MRI artifacts, Diagnostic quality, Susceptibility-weighted imaging

## Abstract

**Objective:**

The aim of this study was to display and quantify signal loss artifacts in 1.5T and 3T brain MRI on a volunteer with different orthodontic appliances.

**Materials and Methods:**

In this experimental study, three different orthodontic appliances were examined on a 1.5T and a 3T MRI scanner in a healthy adult with normal dental occlusion: stainless-steel brackets paired with a nickel-titanium archwire; brackets, archwire, and stainless-steel molar bands; brackets, archwire, molar bands, and a stainless-steel trans-palatal archwire. Assessment of diverse anatomical structures, including different cerebral structures and blood vessels, was conducted using a six-point Likert scale.

**Results:**

Utilizing conventional stainless-steel brackets and a nickel-titanium archwire, with or without the inclusion of stainless-steel molar bands, all cerebral structures demonstrated satisfactory assessability with high diagnostic quality under both 1.5T and 3T MRI. For example, with an average rating of 85/85 for T2 and 77/85 for susceptibility-weighted imaging (SWI). Upon introduction of the stainless-steel trans-palatal archwire, additional artifacts were observed, predominantly manifesting in SWI (20/85), diffusion-weighted imaging (DWI) sequences (31/85), and phase contrast angiography (PCA) (17/20). Differences in artifact severity were mainly observed in the SWI and DWI sequences.

**Conclusion:**

Based on the findings of this study, it is not imperative to entirely remove orthodontic appliances to achieve sufficient diagnostic quality in brain MRI. In instances where SWI or DWI sequences are necessitated, the removal of solely the trans-palatal stainless-steel archwire should be contemplated, given its straightforward execution.

**Clinical Relevance:**

These results highlight the potential to reduce injury risk during orthodontic appliance removal, expedite imaging procedures, and consequently accelerate diagnostic processes, particularly crucial in emergencies.

Magnetic Resonance Imaging (MRI) of the head and brain represents a non-invasive imaging technique devoid of ionizing radiation exposure. MR imaging holds broad applicability, particularly in the diagnostic assessment of central nervous system disorders. In adolescents, this primarily encompasses the identification of inflammatory conditions such as encephalitis and meningitis, tumors, vascular diseases, or trauma [1].

The main magnetic field of an MR scanner imparts substantial forces upon objects composed of metals and alloys exhibiting ferromagnetic characteristics [2]. Multiple metallic implants introduce magnetic field inhomogeneities, resulting in image artifacts [3]. These artifacts manifest as geometric distortions and are discernible as dark voids or conspicuous areas of signal saturation within the images [4]. The severity and dimensions of these artifacts are contingent upon the magnetic properties, spatial alignment, and dimensions of the implants [5, 6].

Within the realm of orthodontics, approximately 30—35% of adolescents require treatment with a fixed orthodontic appliance [7].

Previous investigations have indicated that orthodontic appliances induce artifacts in MRI scans. To preemptively mitigate any diagnostic delays, numerous centers opt for the prophylactic removal of these appliances before conducting MRI examinations. However, the extraction of such orthodontic devices heightens the potential for hard tissue injuries and contributes to an escalation in overall treatment costs. This, in turn, amplifies the financial and biological burdens associated with the procedure [8, 9].

Despite the well-established knowledge that fixed multibracket orthodontic appliances induce image artifacts in MR images, the precise severity and impact on image quality reduction remain uncertain from the present literature [10]. Sadowsky et al. [11] and Hinshaw et al. [12] have observed that artifacts induced by stainless steel brackets and wires do indeed introduce some distortion to MR brain images. However, these distortions typically do not compromise the diagnostic quality of the images. In contrast, several additional studies have substantiated that orthodontic appliances have the potential to render brain MR images non-diagnostic [13, 14].

While 1.5T scanners are commonly employed, 3T scanners have emerged as a prevalent alternative due to their capacity for enhanced image resolution and improved image quality, particularly for neuroimaging. Nevertheless, it is worth noting that 3T scanners may present drawbacks in terms of metal artifacts. The severity of these artifacts amplifies in proportion to the magnetic field strength, thereby prompting discussions concerning the compatibility of orthodontic appliances with both 1.5T and 3T scanners [15].

Zhylich et al. [16] stated that the impact of orthodontic appliances on head MR images varies depending on factors such as the type of appliance, the specific region being imaged, and the applied MR sequences.

The principal objective of the current investigation was to determine if it is possible to adequately assess the brain and the adjacent structures on both, 1.5T and 3T MRI, using three different commonly used fixed orthodontic appliances, attached directly onto the teeth.

## Materials and methods

For the investigations of this self-experiment study, a voluntary healthy 28-year-old female participant was recruited. The subject exhibited normal skeletal and dental characteristics. All 28 permanent teeth were fully erupted, without any metallic restorations or root canal treatments.

The appliances were directly attached to the participant's teeth and scanned consecutively in both a 1.5T and a 3T MRI, respectively. The following fixed orthodontic appliances were examined:

**[Experiment 1]**

Stainless-steel brackets (Bio Quick, Forestadent, Pforzheim, Germany)

+ nickel-titanium archwire 0.014 x 0.025 inch (Forestadent, Pforzheim, Germany)

**[Experiment 2]**

Stainless-steel brackets

+ nickel-titanium archwire 0.014 x 0.025 inch (Forestadent, Pforzheim, Germany)

+ stainless-steel molar bands (Forestadent, Pforzheim, Germany)

**[Experiment 3]**

Stainless-steel brackets

+ nickel-titanium archwire 0.014 x 0.025 inch (Forestadent, Pforzheim, Germany)

+ stainless-steel molar bands (Forestadent, Pforzheim, Germany)

+ stainless-steel trans-palatal archwire (0.032 inch)

The brackets were bonded directly to all the 28 the participant's teeth in the upper and lower jaw using acid-etch technique, following a standardized procedure, and adhering to the manufacturer's instructions. Stainless-steel molar bands were attached to the first molars in the upper jaw on both sides using KetacCem (3M Espe, St Paul, MN, USA). The trans-palatal archwire was previously made by a dental technician and was inserted into the designated palatal locks of the molar bands and secured with a ligature. A 0.014 × 0.025 inch nickel-titanium archwire was conventionally ligated buccally.

### MRI protocol

The examinations were conducted on a 1.5T (MAGNETOM Aera, Siemens Healthineers, Erlangen, Germany) and a 3T (MAGNETOM Skyra, Siemens Healthineers, Erlangen, Germany) whole body MRI system, equipped with a 20-channel phased array head coil. A predefined, standardized protocol for each MRI system was used. Detailed sequence parameters are summarized in Table 1.

**Table 1:** MRI sequence parameters for 1.5T and 3T

	1.5 T									3 T								
Sequence	TE (ms)	TR (ms)	TI (ms)	FA (°)	BW (hz)	SL (mm)	AM	FOV	Imaging plane	TE (ms)	TR (ms)	TI (ms)	FA (°)	BW (Hz)	SL (mm)	AM	FOV	Imaging plane
T2 TSE	90	6800		150	190	2	240 × 320	165*220	tra	96	5300		150	220	2	312 × 384	178*220	tra
T1 MPRAGE	2.69	1960	988	8	160	0.8	272 × 288	217*230	sag	2.12	2060	1040	8	250	0.8	288 × 288	229*229	sag
DWI	97	6100		90	1150	3	128 × 128	220*220	tra	78	6400		90	1565	3	128 × 128	220*220	tra
SWI	40	50		15	80	2.2	192 × 256	165*220	tra	20	28		15	120	1.8	200 × 256	171*220	tra
TIRM dark fluid	95	8000	2370	150	200	3	192 × 256	165*220	cor	84	8500	2440	150	285	3	250 × 320	171*220	cor
TOF	7	27		25	100	0.7	448 × 512	157*180	tra	3,6	22		15	185	0.6	648 × 768	151*180	tra
PCA	9.89	77		15	300	1.1	192 × 256	165*220	tra	7.91	37.75		15	300	3	512 × 512	220*220	tra

*T*, Tesla; *TE*, Echo Time; *TR*, Repitition Time; *TI*, Inversion Time; *FA*, Flip Angle; *BW*, Bandwidth; *SL*, Slice Thickness; *AM*, Acquisition Matrix; *FOV*, Field of View; *T2 TSE*, T2-weighted turbo spin echo sequence; *T1 MPRAGE*, T1 weighted Magnetization Prepared Rapid Gradient Echo; *DWI*, Diffusion weighted imaging; *SWI*, susceptibility-weighted imaging; *TIRM*, Turbo-Inversion Recovery-Magnitude; *TOF*, Time-of-Flight; *PCA*, Phase contrast angiography; *tra*, transversal; *sag*, sagittal; *cor*, coronal;

The following anatomical brain structures were examined: frontal, parietal, temporal, and occipital lobes, basal ganglia, mesencephalon, pons, cerebellum, the third, fourth and lateral ventricles and sella. The orbit, ethmoidal, sphenoidal, and maxillary sinuses, and nasopharynx were studied structures of the facial skull. In addition, the following vessels were assessed: internal carotid artery (ICA), anterior cerebral artery (ACA), middle cerebral artery (MCA), posterior cerebral artery (PCA), vertebral artery (VA), basilar artery (BA), superior sagittal sinus (SSS), transverse sinus (TS), sigmoid sinus (SS), and internal cerebral veins (ICV).

The individual MRI sequences were classified concerning artifact severity and diagnostic quality of the specific anatomical structures by two independent world-certified examiners. The assessment of the different scans was conducted blinded. Image quality was objectively assessed using a 6-point Likert scale.

- Likert 0 = Diagnostics not possible; anatomical structure not visible
- Likert 1 = Poor diagnostic quality; anatomical structure minimally visible
- Likert 2 = Limited diagnostic quality; anatomical structure tolerably visible
- Likert 3 = Moderate diagnostic quality; anatomical structure fairly visible
- Likert 4 = Good diagnostic quality; anatomical structure visible
- Likert 5 = Excellent diagnostic quality; anatomical structure clearly visible

For descriptive statistics, the mean of the individual results from both assessors was calculated and presented in tables. The reader agreement was calculated using Cohen’s Kappa.

## Results

With conventional stainless-steel brackets and a Nickel-Titanium (Ni–Ti) archwire [Experiment 1] in place, comprehensive visibility of all cerebral and vascular structures was achieved, demonstrating excellent clarity in both 1.5T and 3T imaging modalities (see Tables 2 and 3). Facial structures, across most sequences and field strengths, exhibited visibility ranging from moderate to excellent.

**Table 2:**
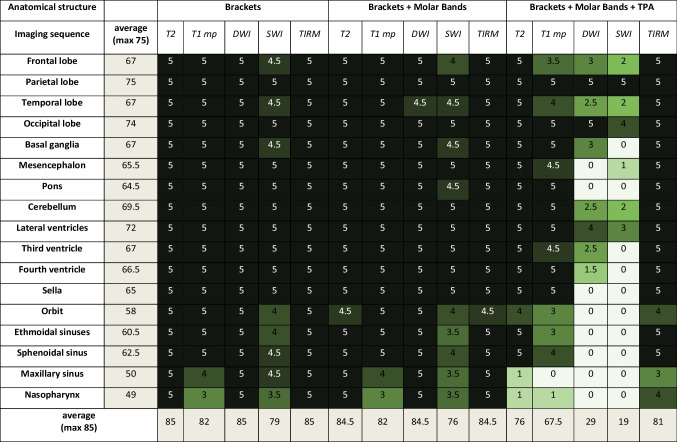
Assessment of artifacts obtained in the 1.5T

Anatomical structure 0: not visible; 1: minimally visible; 2: tolerably visible; 3: fairly visible; 4: visible; 5: clearly visible

**Table 3:**
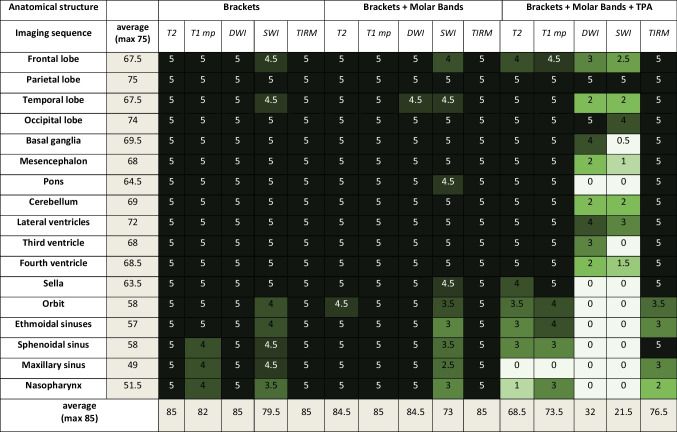
Assessment of artifacts obtained in the 3T scanner

Anatomical structure 0: not visible; 1: minimally visible; 2: tolerably visible; 3: fairly visible; 4: visible; 5: clearly visible

Upon introducing supplementary molar bands [Experiment 2], the findings revealed noteworthy similarities. Furthermore, in coherence with the previously discussed scenario, image quality within the facial sinuses demonstrated a range from moderate to good in Susceptibility-Weighted Imaging (SWI) sequences, observed consistently in both 1.5T and 3T imaging (see Tables 2 and 3).

The incorporation of a trans-palatal archwire [Experiment 3] introduced severe artifacts in both 1.5T and 3T imaging, particularly evident in SWI and Diffusion-Weighted Imaging (DWI) sequences, precluding the evaluation of most anatomical structures. Across various sequences, diagnostic quality for facial sinuses was consistently compromised, with maximal artifacts observed in cerebral structures proximal to the skull base (see Tables 2 and 3).

**Fig. 1:**
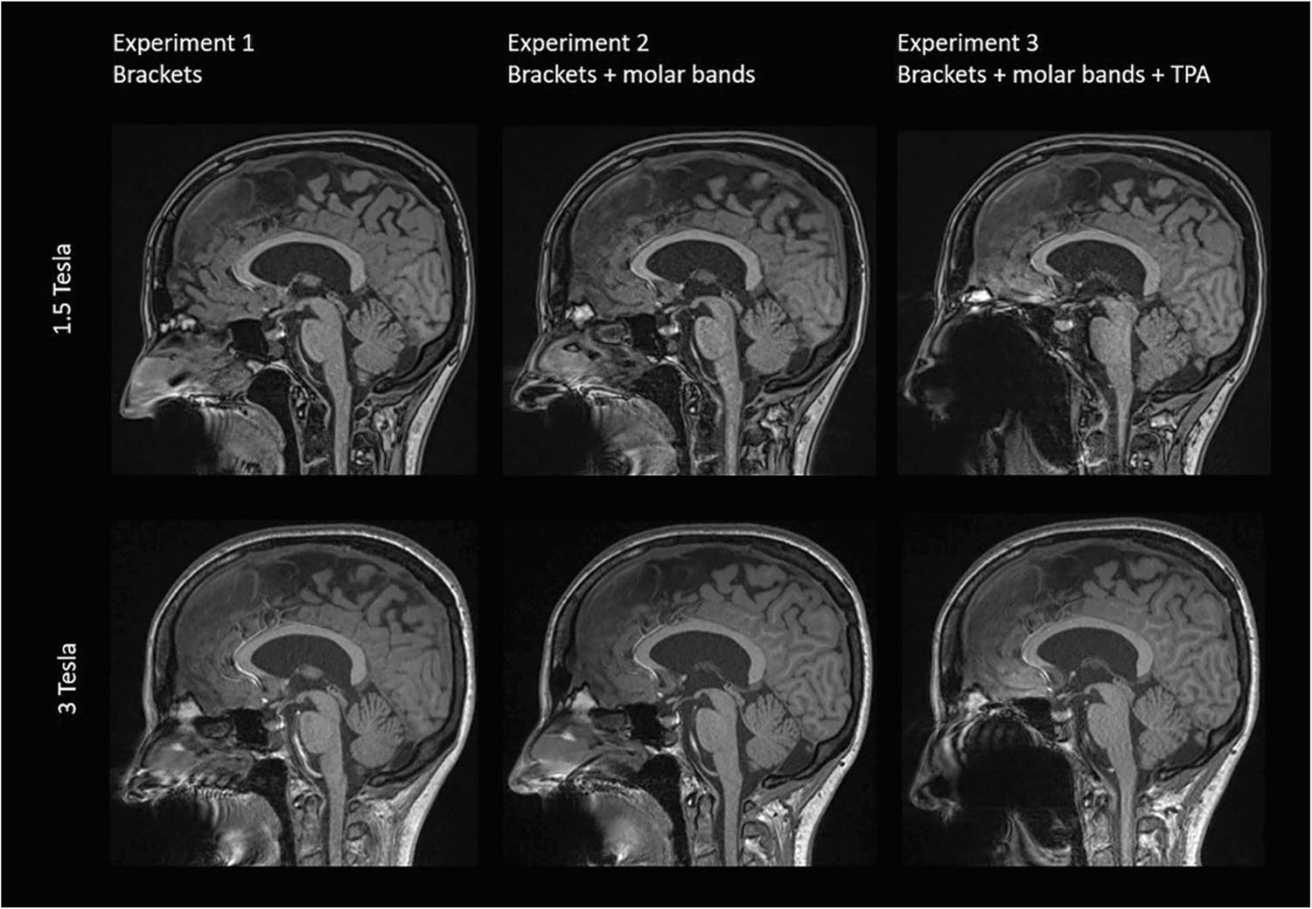
Comparison of representative slices of a sagittal T1 weighted image (MPRAGE) from all three volunteer Experiments acquired at 1.5T and 3T

Figure 1 shows representative sagittal T1 weighted images acquired at 1.5T (top row) and 3T (bottom row) for all three experiments. The strongest artefacts occur after introducing supplementary molar bands [Experiment 3].

**Fig. 2:**
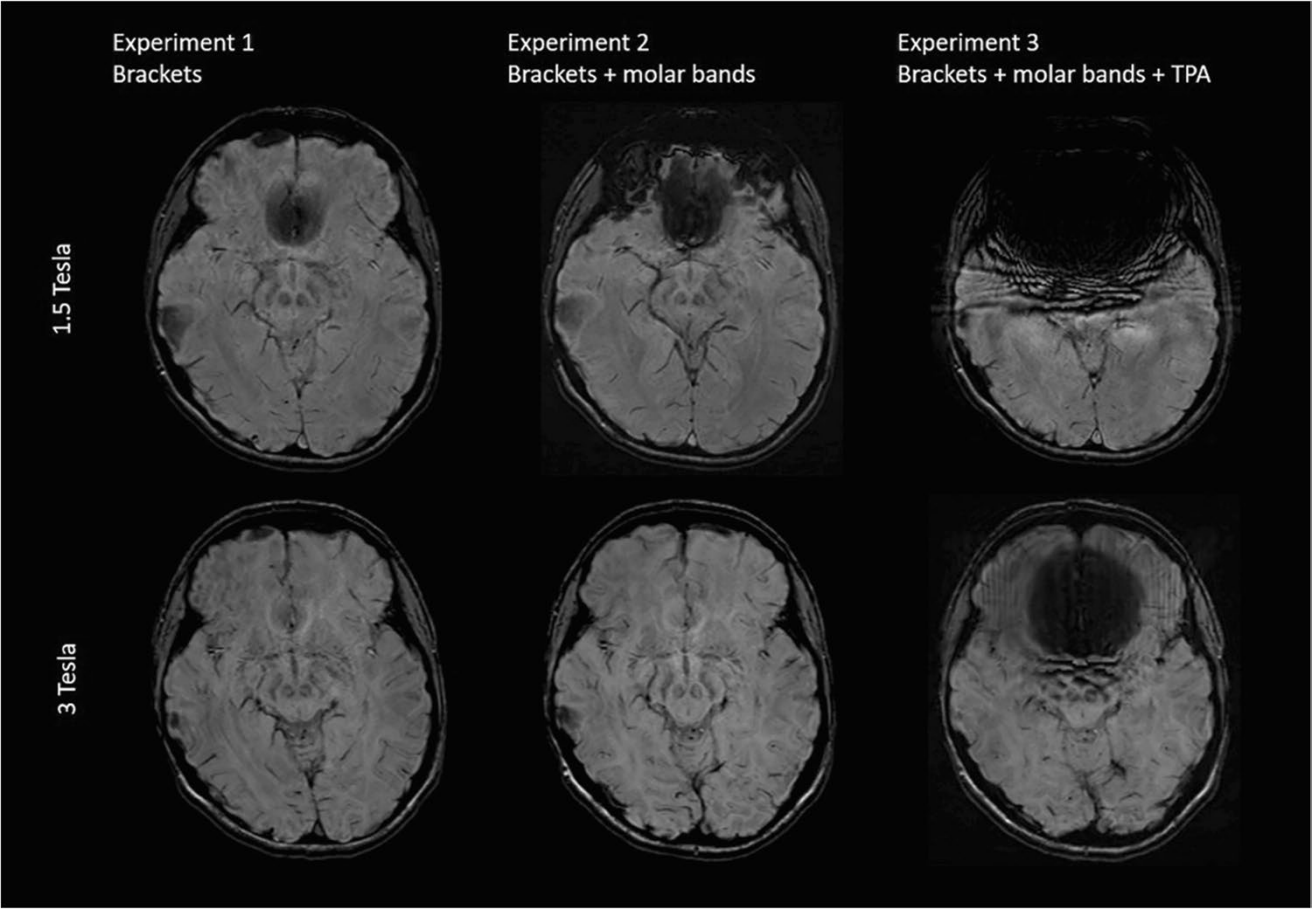
Comparison of representative slices of an axial SWI image from all three volunteer Experiments acquired at 1.5T and 3T

**Fig. 3:**
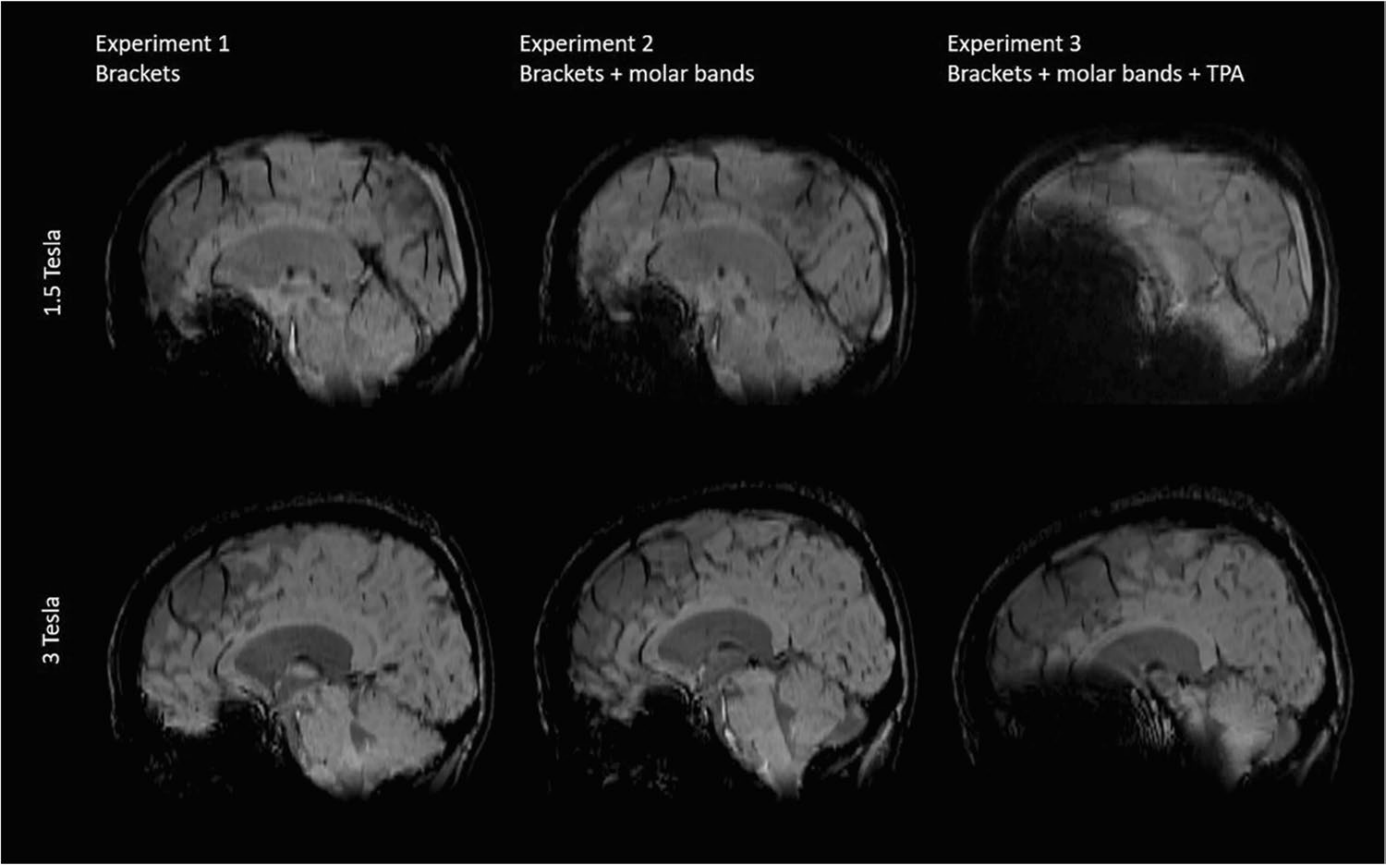
Comparison of representative slices of a reconstructed sagittal SWI image from all three volunteer Experiments acquired at 1.5T and 3T

The strongest artifacts are observed in SWI as shown in Figs. 2 and 3, respectively.

Tables 4 and 5 show the assessment of artifacts in the vascular system due to the three different orthodontic appliances obtained in the 1.5T and 3T scanner. It has been observed that the diagnostic quality of cerebral veins was restricted in Phase Contrast Angiography (PCA), specifically at a field strength of 3T (see Table 5).

**Table 4:**
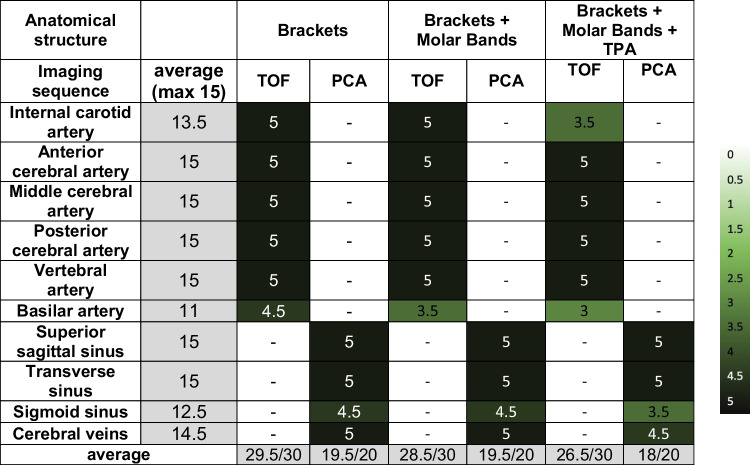
Assessment of artifacts in the vascular system due to brackets obtained in the 1.5-Tesla scanner

Anatomical structure 0: not visible; 1: minimally visible; 2: tolerably visible; 3: fairly visible; 4: visible; 5: clearly visible

**Table 5:**
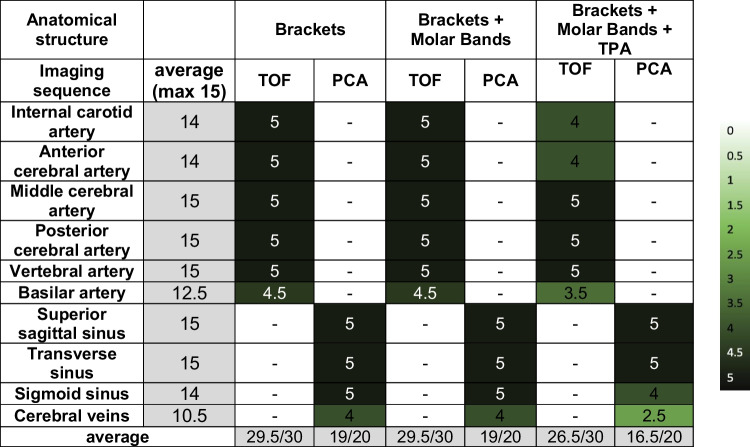
Assessment of artifacts in the vascular system due to brackets obtained in the 3-Tesla scanner

Anatomical structure 0: not visible; 1: minimally visible; 2: tolerably visible; 3: fairly visible; 4: visible; 5: clearly visible

We observed a high agreement between the two raters across both field strengths and different orthodontic appliances (kappa = 0.926, *p* < 0.001).

## Discussion

To the best of our knowledge, this study represents the first exploration of the extent of artifacts in brain MRI, caused by commonly used orthodontic appliances in MRI with different filed strengths.

The present investigation demonstrates that the assessment of all cerebral and vascular structures was mostly excellent in the 1.5T and 3T MRI scanner and in all sequences using only conventional brackets, Ni–Ti archwire and molar bands. When adding a trans-palatal archwire, brain structures in all sequences could be assessed to at least a moderate level except for the SWI and DWI sequence. The diagnostic quality of cerebral veins in phase-contrast-angiography was restricted to the 3T MRI.

We can conclude that the evaluation of the anatomical structures of interest, was at least moderately assessable on T2 weighted, T1 MPRAGE, FLAIR and TOF images, for each orthodontic appliance. This suggests that sufficient structural image analysis of the brain can be performed initially with all orthodontic appliances [17, 18]. The ability to evaluate T1 (MPRAGE) sequences allows administration of contrast agents, which greatly improves visualization of blood–brain-barrier [19] and primary or metastatic brain tumors [17]. Adequate evaluation of T2 weighted images and of FLAIR in combination with the T1-weighted sequences can clarify the questions of inflammation such as encephalitis and meningitis [20–22]. Preliminary assessment for possible vascular pathogenesis such as intracranial occlusions, intracranial aneurysm, and vascular malformations of the central nervous system is also feasible by adding the TOF sequence [23, 24] with all used orthodontic appliances included a stainless-steel trans-palatal archwire.

Another determining factor in the generation of metal-related artifacts during MRI imaging is the selected pulse sequence, which relates to the type and severity of metal-related artifacts. Spin-echo (SE) sequences employ 180° radiofrequency pulses for refocusing, which serve to correct static or fixed magnetic field inhomogeneities. In contrast, Gradient echo (GRE) sequences use gradient fields for refocusing, making them more prone to field inhomogeneities. The predominant reason for signal loss in GRE imaging is intravoxel dephasing [25]. Examining metal-ceramic restorations revealed a notable distinction between images obtained through GRE and SE pulse sequences [26]. GRE images display significantly more artifacts compared to SE [25–27].

SWI and DWI sequences are sensitive to metal artifacts: SWI sequence is a GRE sequence and is therefore particularly sensitive to compounds that distort the local magnetic field [28]. Furthermore, for SWI, the magnitude and phase of the GRE sequence are combined which further increase the sensitivity for metal induced artifacts. The DWI sequence is very sensitive to changes in water diffusion for which strong diffusion sensitive gradients are required. DWI sequence are strongly associated with the occurrence of image distortion caused by susceptibility shifts [13, 29].

SWI technique is used for depiction of intraparenchymal hemorrhages [30], differentiation of late-stage blood products [31, 32] as well as of internal vascular architecture [17]. For traumatic brain injuries, the SWI technique stands out as more effective than other approaches when it comes to identifying and characterizing the quantity, dimensions, volume, and spatial distribution of hemorrhagic lesions commonly observed in cases of diffuse axonal injury, particularly those involving the brainstem [33–35]. DWI sequences are applied in suspected acute ischemic stroke [36], as it has the capability to reveal ischemic changes as early as 11 min following the onset of a stroke [37]. Besides, DWI is widely employed in oncological contexts, serving as a tool for evaluating treatment responses and monitoring the disease progression, and for detecting white matter diseases [38]. When it comes to diagnosing acute signs of stroke or intracranial hemorrhage, the DWI and SWI sequences are the method of choice. Phase-contrast magnetic resonance angiography (PC MRA) serves the purpose of observing the flow of both arteries and veins. It is employed to assess conditions like intracranial occlusions, intracranial aneurysms, and vascular malformations [39]. Notably, no contrast agent is required, and it offers advantages in scenarios involving hemorrhaging compared to the TOF technique [39, 40]. Conversely, the extended data acquisition time may increase vulnerability to motion artifacts [41].

Based on our self-experimentation, we suggest that when evaluating acute signs of stroke or intracranial hemorrhage through MRI, it is challenging to assess indications accurately in the presence of an orthodontic appliance with a stainless-steel trans-palatal archwire, even at 1.5T. Nevertheless, we found that interpreting images exclusively with brackets and molar bands provides sufficient clarity for assessment at both 1.5T and 3T. In cases unrelated to stroke or intracranial hemorrhage, common indications can be effectively evaluated without requiring the use of SWI and DWI sequences. This implies that there is no necessity to remove brackets, molar bands, or the trans-palatal archwire for the assessment of these common indications.

The selection of radiological imaging modality in the head region primarily depends on the clinical question, the ability of the chosen method to visualize the relevant structures, and its availability. Ultrasound is often the ideal and superior technique for evaluating superficial soft tissues, such as lymph nodes, salivary glands, and cutaneous structures. It is also useful for the acute diagnosis of orbital conditions. Specialized exams like angiography or sialography are minimally affected by dental implants. The advantage of Cone Beam Computed Tomography (CBCT) over conventional Computed Tomography (CT) for visualizing teeth and jaws lies in its higher resolution, while CT is better suited for imaging soft tissues. Generally, CT has long been known for its shorter acquisition times and broader availability compared to MRI. It reliably detects intracranial hemorrhages, for instance, and offers high-resolution images of bone structures, while MRI excels in soft tissue contrast but is more time-consuming [42]. Today, metal artifacts can be significantly reduced with advanced techniques such as dual-energy CT [43] or photon-counting detector CT [44]. CT of the skull is recommended as an initial diagnostic test in children suspected of child abuse [45]. However, whenever possible, MRI should be preferred for imaging the brain in pediatric cases, as it can detect not only intracranial trauma sequelae but also ocular hemorrhage in cases of child abuse [46]. Moreover, it avoids radiation exposure. Intracranial MRI is clearly the preferred modality for imaging hemorrhages, tumors, inflammatory diseases, or vascular pathologies.

One notable advantage of this study is the explicit comparison between images acquired on a 1.5T and a 3T MRI system. No substantial differences in artifact severity were observed between the field-strengths in Experiment 1 and 2, enabling the utilization of any MRI system based on its availability. However, our results show that in Experiment 3, more artifacts occurred at 1.5T, particularly in the T1 MPRAGE, DWI, and SWI sequences. In contrast, the T2 and TIRM sequences at 1.5T exhibit fewer artifacts in Experiment 3.

Stronger artifacts in SWI at 1.5T are likely due to the thicker slices (2.2 mm at 1.5T vs. 1.8 mm at 3T), a lower bandwidth (80 Hz at 1.5T vs. 120 Hz at 3T) and longer echo time (40 ms at 1.5T vs. 20 ms at 3T). All these factors together will favor stronger artifacts at 1.5T compared to 3T. Furthermore, also the T1 MPRAGE and DWI sequence have lower band with at 1.5T compared to 3T, which make them more prone to artifacts.

Although both SWI and DWI sequences, which exhibited the most artifacts, were acquired in a transverse image orientation, there is no definitive evidence to establish a clear association between imaging plane and artifact occurrence.

The interactions between orthodontic appliances and MRI have been extensively studied, with particular focus on thermal effects and potential impacts on bracket stability. Recent research indicates that these concerns are largely unwarranted for most orthodontic appliances. Thermal effects due to radiofrequency heating during MRI have been found to be minimal, with temperature increases generally well below the threshold for tissue damage or patient discomfort [47, 48]. Studies have shown that the temperature increase in metallic brackets and wires is clinically insignificant and typically ranging from 0.05 °C to 2.4 °C for brackets and 0.42 °C to 1.74 °C for wires [49]. Even at a magnetic field strength of 3T, the temperature rise remains modest, with mean increases of about 1–2 °C [49]. This low level of heating poses negligible risk to patients and does not compromise the integrity of the orthodontic appliances or their mounting to teeth [49, 50]. Overall, these findings support the conclusion that patients with orthodontic appliances can safely undergo MRI in most cases, though individual assessment remains advisable [47, 51].

It is important to note, that we used clinical sequences, which are optimized to generate the best image contrast for each field strength rather that optimized for minimal artifact occurrence. Additionally, the volunteer did not experience changes in temperature or feelings of traction on the implants during the MRI scans.

Limitation: Only three specific dental appliance conditions were examined solely in one individual without a known pathology in one of the investigated structures. Further investigation needs to be conducted in a larger cohort and with different orthodontic appliances are needed, to confirm the general validity.

## Conclusion

In conclusion, the necessity to remove any orthodontic appliance prior to MR imaging is generally unwarranted for most clinical examinations. In particular, structural T1 weighted, T2 weighted or FLAIR sequences can sufficiently be used with orthodontic appliances. In instances of acute indications such as stroke or intracranial hemorrhage, where DWI and SWI sequences are required, the removal of the stainless-steel palatal archwire alone proves sufficient. Nonetheless, specific decisions should be made with consideration for the targeted anatomical region, the clinical indication, required imaging sequences, and the type of orthodontic appliance in use.

## Data Availability

No datasets were generated or analysed during the current study.

## References

[1] Mehan WA Jr, Gonzalez RG, Buchbinder BR, Chen JW, Copen WA, Gupta R et al (2014) Optimal brain MRI protocol for new neurological complaint. PLoS ONE 9(10):e110803. 10.1371/journal.pone.0110803 25343371 10.1371/journal.pone.0110803 PMC4208779

[2] Niraj LK, Patthi B, Singla A, Gupta R, Ali I, Dhama K et al (2016) MRI in Dentistry- A Future Towards Radiation Free Imaging - Systematic Review. J Clin Diagn Res 10(10):ZE14–ZE19. 10.7860/JCDR/2016/19435.8658 27891491 10.7860/JCDR/2016/19435.8658 PMC5121829

[3] Hargreaves BA, Worters PW, Pauly KB, Pauly JM, Koch KM, Gold GE (2011) Metal-induced artifacts in MRI. AJR Am J Roentgenol 197(3):547–555. 10.2214/AJR.11.7364 21862795 10.2214/AJR.11.7364 PMC5562503

[4] Beuf O, Lissac M, Cremillieux Y, Briguet A (1994) Correlation between magnetic resonance imaging disturbances and the magnetic susceptibility of dental materials. Dent Mater 10(4):265–268. 10.1016/0109-5641(94)90072-8 7664995 10.1016/0109-5641(94)90072-8

[5] Fache JS, Price C, Hawbolt EB, Li DK (1987) MR imaging artifacts produced by dental materials. AJNR Am J Neuroradiol 8(5):837–840 3118677 PMC8334505

[6] Shellock FG, Kanal E (1998) Aneurysm clips: evaluation of MR imaging artifacts at 1.5 T. Radiology 209(2):563–566. 10.1148/radiology.209.2.9807590 9807590 10.1148/radiology.209.2.9807590

[7] Dimberg L, Lennartsson B, Arnrup K, Bondemark L (2015) Prevalence and change of malocclusions from primary to early permanent dentition: a longitudinal study. Angle Orthod 85(5):728–734. 10.2319/080414-542.1 25867255 10.2319/080414-542.1 PMC8610411

[8] Wylezinska M, Pinkstone M, Hay N, Scott AD, Birch MJ, Miquel ME (2015) Impact of orthodontic appliances on the quality of craniofacial anatomical magnetic resonance imaging and real-time speech imaging. Eur J Orthod 37(6):610–617. 10.1093/ejo/cju103 25667040 10.1093/ejo/cju103

[9] Beau A, Bossard D, Gebeile-Chauty S (2015) Magnetic resonance imaging artefacts and fixed orthodontic attachments. Eur J Orthod 37(1):105–110. 10.1093/ejo/cju020 24997025 10.1093/ejo/cju020

[10] Harris EF (2001) Effects of patient age and sex on treatment: correction of Class II malocclusion with the Begg technique. Angle Orthod 71(6):433–441. 10.1043/0003-3219(2001)071%3c0433:EOPAAS%3e2.0.CO;2 11771781 10.1043/0003-3219(2001)071<0433:EOPAAS>2.0.CO;2

[11] Sadowsky PL, Bernreuter W, Lakshminarayanan AV, Kenney P (1988) Orthodontic appliances and magnetic resonance imaging of the brain and temporomandibular joint. Angle Orthod 58(1):9–20. 10.1043/0003-3219(1988)058%3c0009:OAAMRI%3e2.0.CO;2 3162666 10.1043/0003-3219(1988)058<0009:OAAMRI>2.0.CO;2

[12] Hinshaw DB Jr, Holshouser BA, Engstrom HI, Tjan AH, Christiansen EL, Catelli WF (1988) Dental material artifacts on MR images. Radiology 166(3):777–779. 10.1148/radiology.166.3.3340777 3340777 10.1148/radiology.166.3.3340777

[13] Elison JM, Leggitt VL, Thomson M, Oyoyo U, Wycliffe ND (2008) Influence of common orthodontic appliances on the diagnostic quality of cranial magnetic resonance images. Am J Orthod Dentofacial Orthop 134(4):563–572. 10.1016/j.ajodo.2006.10.038 18929275 10.1016/j.ajodo.2006.10.038

[14] Okano Y, Yamashiro M, Kaneda T, Kasai K (2003) Magnetic resonance imaging diagnosis of the temporomandibular joint in patients with orthodontic appliances. Oral Surg Oral Med Oral Pathol Oral Radiol Endod 95(2):255–263. 10.1067/moe.2003.37 12582369 10.1067/moe.2003.37

[15] Olsrud J, Latt J, Brockstedt S, Romner B, Bjorkman-Burtscher IM (2005) Magnetic resonance imaging artifacts caused by aneurysm clips and shunt valves: dependence on field strength (1.5 and 3 T) and imaging parameters. J Magn Reson Imaging 22(3):433–437. 10.1002/jmri.20391 16104008 10.1002/jmri.20391

[16] Zhylich D, Krishnan P, Muthusami P, Rayner T, Shroff M, Doria A et al (2017) Effects of orthodontic appliances on the diagnostic quality of magnetic resonance images of the head. Am J Orthod Dentofacial Orthop 151(3):484–499. 10.1016/j.ajodo.2016.07.020 28257733 10.1016/j.ajodo.2016.07.020

[17] Villanueva-Meyer JE, Mabray MC, Cha S (2017) Current Clinical Brain Tumor Imaging. Neurosurgery 81(3):397–415. 10.1093/neuros/nyx103 28486641 10.1093/neuros/nyx103 PMC5581219

[18] Yamamoto J, Kakeda S, Shimajiri S, Nakano Y, Saito T, Ide S et al (2018) Evaluation of peritumoral brain parenchyma using contrast-enhanced 3D fast imaging employing steady-state acquisition at 3T for differentiating metastatic brain tumors and glioblastomas. World Neurosurg 120:e719–e729. 10.1016/j.wneu.2018.08.147 30165229 10.1016/j.wneu.2018.08.147

[19] Gale EM, Caravan P (2018) Gadolinium-free contrast agents for magnetic resonance imaging of the central nervous system. ACS Chem Neurosci 9(3):395–397. 10.1021/acschemneuro.8b00044 29431424 10.1021/acschemneuro.8b00044 PMC5994911

[20] Jayaraman K, Rangasami R, Chandrasekharan A (2018) Magnetic resonance imaging findings in viral encephalitis: a pictorial essay. J Neurosci Rural Pract 9(4):556–560. 10.4103/jnrp.jnrp_120_18 30271050 10.4103/jnrp.jnrp_120_18 PMC6126294

[21] Carmo RLD, Alves Simao AK, Amaral L, Inada BSY, Silveira CF, Campos CMS et al (2019) Neuroimaging of Emergent and Reemergent Infections. Radiographics 39(6):1649–1671. 10.1148/rg.2019190020 31589575 10.1148/rg.2019190020

[22] Albrecht DS, Granziera C, Hooker JM, Loggia ML (2016) In vivo imaging of human neuroinflammation. ACS Chem Neurosci 7(4):470–483. 10.1021/acschemneuro.6b00056 26985861 10.1021/acschemneuro.6b00056 PMC5433433

[23] Tang H, Hu N, Yuan Y, Xia C, Liu X, Zuo P et al (2019) Accelerated time-of-flight magnetic resonance angiography with sparse undersampling and iterative reconstruction for the evaluation of intracranial arteries. Korean J Radiol 20(2):265–274. 10.3348/kjr.2017.0634 30672166 10.3348/kjr.2017.0634 PMC6342758

[24] Martin-Noguerol T, Concepcion-Aramendia L, Lim CT, Santos-Armentia E, Cabrera-Zubizarreta A, Luna A (2021) Conventional and advanced MRI evaluation of brain vascular malformations. J Neuroimaging 31(3):428–445. 10.1111/jon.12853 33856735 10.1111/jon.12853

[25] Stradiotti P, Curti A, Castellazzi G, Zerbi A (2009) Metal-related artifacts in instrumented spine. Techniques for reducing artifacts in CT and MRI: state of the art. Eur Spine J 18(Suppl 1):102–108. 10.1007/s00586-009-0998-5 19437043 10.1007/s00586-009-0998-5 PMC2899595

[26] Cortes AR, Abdala-Junior R, Weber M, Arita ES, Ackerman JL (2015) Influence of pulse sequence parameters at 1.5 T and 3.0 T on MRI artefacts produced by metal-ceramic restorations. Dentomaxillofac Radiol 44(8):20150136. 10.1259/dmfr.20150136 26084475 10.1259/dmfr.20150136 PMC4628425

[27] Klinke T, Daboul A, Maron J, Gredes T, Puls R, Jaghsi A et al (2012) Artifacts in magnetic resonance imaging and computed tomography caused by dental materials. PLoS ONE 7(2):e31766. 10.1371/journal.pone.0031766 22384071 10.1371/journal.pone.0031766 PMC3285178

[28] Haller S, Haacke EM, Thurnher MM, Barkhof F (2021) Susceptibility-weighted imaging: technical essentials and clinical neurologic applications. Radiology 299(1):3–26. 10.1148/radiol.2021203071 33620291 10.1148/radiol.2021203071

[29] Werner S, Zinsser D, Esser M, Nickel D, Nikolaou K, Othman AE (2023) Enhanced image processing using complex averaging in diffusion-weighted imaging of the prostate: the impact on image quality and lesion detectability. Diagnostics (Basel) 13(14), 10.3390/diagnostics13142325 10.3390/diagnostics13142325 PMC10378377 37510071

[30] Borrega-Mouquinho Y, Sanchez-Gomez J, Fuentes-Garcia JP, Collado-Mateo D, Villafaina S (2021) Effects of high-intensity interval training and moderate-intensity training on stress, depression, anxiety, and resilience in healthy adults during coronavirus disease 2019 confinement: a randomized controlled trial. Front Psychol 12:643069. 10.3389/fpsyg.2021.643069 33716913 10.3389/fpsyg.2021.643069 PMC7943442

[31] Jain N, Kumar S, Singh A, Jain S, Phadke RV (2023) Blood in the brain on susceptibility-weighted imaging. Indian J Radiol Imaging 33(1):89–97. 10.1055/s-0042-1758880 36855723 10.1055/s-0042-1758880 PMC9968548

[32] Weerink LB, Appelman AP, Kloet RW, Van der Hoorn A (2023) Susceptibility-weighted imaging in intracranial hemorrhage: not all bleeds are black. Br J Radiol 96(1148):20220304. 10.1259/bjr.20220304 35766940 10.1259/bjr.20220304 PMC10392652

[33] Tong KA, Ashwal S, Holshouser BA, Shutter LA, Herigault G, Haacke EM et al (2003) Hemorrhagic shearing lesions in children and adolescents with posttraumatic diffuse axonal injury: improved detection and initial results. Radiology 227(2):332–339. 10.1148/radiol.2272020176 12732694 10.1148/radiol.2272020176

[34] Halefoglu AM, Yousem DM (2018) Susceptibility weighted imaging: Clinical applications and future directions. World J Radiol 10(4):30–45. 10.4329/wjr.v10.i4.30 29849962 10.4329/wjr.v10.i4.30 PMC5971274

[35] Mittal S, Wu Z, Neelavalli J, Haacke EM (2009) Susceptibility-weighted imaging: technical aspects and clinical applications, part 2. AJNR Am J Neuroradiol 30(2):232–252. 10.3174/ajnr.A1461 19131406 10.3174/ajnr.A1461 PMC3805373

[36] Kakkar P, Kakkar T, Patankar T, Saha S (2021) Current approaches and advances in the imaging of stroke. Dis Model Mech 14(12). 10.1242/dmm.048785 10.1242/dmm.048785 PMC8669490 34874055

[37] Hjort N, Christensen S, Solling C, Ashkanian M, Wu O, Rohl L et al (2005) Ischemic injury detected by diffusion imaging 11 minutes after stroke. Ann Neurol 58(3):462–465. 10.1002/ana.20595 16130095 10.1002/ana.20595

[38] Baliyan V, Das CJ, Sharma R, Gupta AK (2016) Diffusion weighted imaging: technique and applications. World J Radiol 8(9):785–798. 10.4329/wjr.v8.i9.785 27721941 10.4329/wjr.v8.i9.785 PMC5039674

[39] Wymer DT, Patel KP, Burke WF 3rd, Bhatia VK (2020) Phase-contrast MRI: physics, techniques, and clinical applications. Radiographics 40(1):122–140. 10.1148/rg.2020190039 31917664 10.1148/rg.2020190039

[40] Oura D, Ihara R, Myo E, Sato S, Sugimori H (2022) Construction of super-rapid brain MRA using oblique transverse acquisition phase contrast angiography with tilted optimized non-saturated excitation pulse. Magn Reson Imaging 85:193–201. 10.1016/j.mri.2021.10.037 34715289 10.1016/j.mri.2021.10.037

[41] Kuo AH, Nagpal P, Ghoshhajra BB, Hedgire SS (2019) Vascular magnetic resonance angiography techniques. Cardiovasc Diagn Ther 9(Suppl 1):28–36. 10.21037/cdt.2019.06.07 10.21037/cdt.2019.06.07 PMC6732109 31559152

[42] Laughlin S, Montanera W (1998) Central nervous system imaging. When is CT more appropriate than MRI? Postgrad Med 104:73–88. 10.3810/pgm.1998.11.402 9823386 10.3810/pgm.1998.11.402

[43] Katsura M, Sato J, Akahane M, Kunimatsu A, Abe O (2018) Current and novel techniques for metal artifact reduction at CT: practical guide for radiologists. Radiographics 38:450–461. 10.1148/rg.2018170102 29528826 10.1148/rg.2018170102

[44] Baffour FI, Glazebrook KN, Ferrero A, Leng S, McCollough CH, Fletcher JG, Rajendran K (2023) Photon-counting detector CT for musculoskeletal imaging: a clinical perspective. AJR Am J Roentgenol 220:551–560. 10.2214/AJR.22.28418 36259593 10.2214/AJR.22.28418

[45] Colleran GC, Fossmark M, Rosendahl K, Argyropoulou M, Mankad K, Offiah AC (2024) ESR essentials: imaging of suspected child abuse-practice recommendations by the European society of paediatric radiology. Eur Radiol. 10.1007/s00330-024-11052-4 39289300 10.1007/s00330-024-11052-4 PMC11914366

[46] Teixeira SR, Gonçalves FG, Servin CA, Mankad K, Zuccoli G (2018) Ocular and intracranial mr imaging findings in abusive head trauma. Top Magn Reson Imaging 27:503–514. 10.1097/RMR.0000000000000169 30516697 10.1097/RMR.0000000000000169

[47] Dobai A, Dembrovszky F, Vízkelety T, Barsi P, Juhász F, Dobó-Nagy C (2022) MRI compatibility of orthodontic brackets and wires: systematic review article. BMC Oral Health 22:298. 10.1186/s12903-022-02317-9 35854295 10.1186/s12903-022-02317-9 PMC9295293

[48] Regier M, Kemper J, Kaul MG, Feddersen M, Adam G, Kahl-Nieke B, Klocke A (2009) Radiofrequency-induced heating near fixed orthodontic appliances in high field mri systems at 3.0 tesla. J Orofac Orthop 70:485–494. 10.1007/s00056-009-9923-0 19960291 10.1007/s00056-009-9923-0

[49] Sfondrini MF, Preda L, Calliada F, Carbone L, Lungarotti L, Bernardinelli L, Gandini P, Scribante A (2019) Magnetic resonance imaging and its effects on metallic brackets and wires: does it alter the temperature and bonding efficacy of orthodontic devices? Materials (Basel) 12:3971. 10.3390/ma12233971 31801202 10.3390/ma12233971 PMC6926903

[50] Sfondrini MF, Gallo S, Pascadopoli M, Rizzi C, Boldrini A, Santagostini S, Anemoni L, Gorone MSP, Preda L, Gandini P (2023) Effect of magnetic resonance imaging at 1.5 T and 3 T on temperature and bond strength of orthodontic bands with welded tubes: an in vitro study. Materials (Basel) 16:651. 10.3390/ma16020651 36676387 10.3390/ma16020651 PMC9863444

[51] Chockattu SJ, Suryakant DB, Thakur S (2018) Unwanted effects due to interactions between dental materials and magnetic resonance imaging: a review of the literature. Restor Dent Endod 43:e39. 10.5395/rde.2018.43.e39 30483463 10.5395/rde.2018.43.e39 PMC6237727

